# Food Neophobia and Food Disgust: The Mediating Role of Perceived Vulnerability to Disease

**DOI:** 10.3390/bs11050065

**Published:** 2021-04-29

**Authors:** Giuseppe Santisi, Paola Magnano, Vittorio Edoardo Scuderi

**Affiliations:** 1 Department of Educational Sciences, University of Catania, Via Biblioteca 4, 95124 Catania, Italy; vittorioscuderi9620@gmail.com; 2 Faculty of Human and Social Sciences, Kore University, Cittadella Universitaria, 94100 Enna, Italy; paola.magnano@unikore.it

**Keywords:** food neophobia, food disgust, eating behavior, perceived vulnerability to disease, psychological attitudes

## Abstract

Negative attitudes towards food are influenced by two factors, neophobia and often related disgust. Neophobia is the tendency to avoid new foods, while food disgust is the refusal of food that is considered potentially harmful to health. The study presented here aims to analyze the correlation between these two attitudes and the possible mediation operated by the perception of vulnerability to diseases, in order to understand if and how this contributes to the disgust towards certain unfamiliar foods. The study was developed through the administration of an anonymous questionnaire to a sample of 487 Italian citizens participating on a voluntary basis. Three tools were used: Food Neophobia Scale, Perceived Vulnerability to Disease, and Food Disgust Scale. The results showed a strong positive correlation between food disgust and food neophobia. Furthermore, through the application of structural mediation models, it has been shown that between food neophobia and food disgust, there is a mediation effect determined by perceived infectivity. The research aims to make a significant contribution to the understanding of the relationship between food disgust and some individual and psychological characteristics of people, demonstrating that the fear of disease transmission affects their food choices.

## 1. Introduction

The Italian large-scale distribution market in the agri-food sector shows some interesting trends: 2021 is considered the year of sober food, with the confirmation of a strategy based on the concept of “slow cooking”: on the one hand, more purchases of basic ingredients and fewer ready meals; on the other hand, the defense of the quality and healthiness of food produced with local, natural, and sustainable raw materials; production, that is, linked to the territory and to controlled supply chains [1].

Eating behavior is therefore still essential for the individual and the cultural heritage of their community. In a global situation characterized by profound uncertainties and fears of a contingent nature and by an ever-increasing push towards environmental sustainability objectives, what reactions are recorded towards the presence of new foods?

Food consumption has to cope with one of the aspects that both humans and rats have in common, which is neophobia [2]. Food neophobia has been defined as “a reluctance to eat and/or avoidance of novel foods” [3] (p. 105). It is important to remember the omnivore’s dilemma [4] which highlights the fact that animals must both approach and avoid novel foods. Animals have developed adaptive behaviors to satisfy their energy and nutrient needs while avoiding potential toxins by choosing foods that provide benefits over those perceived as unsafe [5]. Accordingly, the smell and taste systems have direct connections to the hypothalamus and limbic system which control the emotional responses that lead to strong attraction and aversion towards tastes and smells of food and beverages [6]. Supposedly, there are several factors that lead to a resolution of the conflict, leading the individual to opt for or avoid a particular food at any specific time [3]. Several studies showed that there are people who are particularly inclined to broaden their diet to introduce different and unfamiliar foods while others show a strong reluctance and concern when including new foods in their diet [7].

Animal foods represent a high risk due to the possible transmission of pathogens. Although cooking meat can significantly reduce this risk, it is necessary that the meat is cooked properly, and the equipment used is disinfected. For this reason, human and non-human beings have developed psychological mechanisms for caution towards meat consumption [8]. Consequently, the emotion of disgust is aimed at avoiding the threat of infectious diseases in general from food [9,10] and it is perceived as a sense of repulsion and nausea that leads to a strong desire to turn away from the stimulus that has aroused this feeling [11]. Food disgust has been defined as a “food-rejection emotion intended to prevent the ingestion of potentially noxious and/or pathogen-laden substances” [12] (p. 3). Despite being connected to pathogen avoidance, it is necessary to be aware that the willingness to eat unfamiliar food is also linked to various social dynamics (economic, cultural, ethnic, and religious) together with beliefs, values, motivations, and socioeconomic conditions [13,14]. Several researchers have shown that food neophobia is closely related to food disgust [2,15,16] but without deepening the role of perceived vulnerability to disease between both dimensions. The perceived vulnerability to disease can be distinguished as perceived infectability, which is related to the individual’s beliefs of being vulnerable to health issues (i.e., infectious diseases) in the future and germ aversion, which is defined as emotional distress in situations entailing an elevated risk of disease transmission. Specifically, the first dimension refers to perceived susceptibility to contagious diseases based on an individual’s subjective beliefs, while the second reflects objective individual discomfort related to the increased chance of pathogen transmission in specific circumstances [17]. It has been suggested that the relationship between the perception of vulnerability to disease and health preferences could lead to people’s sensitivity to all types of stimuli perceived as unpleasant [17]. Many phenomena in the area of social cognition are conditioned by different subjective perceptions of diseases and the possibility of contracting them [18]. Furthermore, disgust has been shown to have a disease avoidance function in humans through physical and non-physical stimulation [19]. As the pathogenic aspects of food lead individuals to be more cautious and conscientious, it is necessary to investigate individuals’ differences in perception of vulnerability to disease in order to understand if and how it contributes to disgust towards certain unfamiliar foods.

## 2. Theoretical Background

Several studies have shown that disgust plays a key role in causing rejection behavior and distancing from foods considered potentially dangerous or containing pathogens [10,12,18,19]. Rozin Milman and Nwmeroff [20] classified four food rejection forms: distaste, which is based on negative sensory perceptions; danger, motivated by the prevention of being subjected to physical repercussions; inappropriate, an assessment of what should be ingested in terms of ideological and cultural reasons; disgust, which considers an item’s origin or the social historical background of it. Subsequently, Rozin, Haidt, and McCauley [11] provided the RHM (Rozin-Haidt-McCauley) theoretical model which is based on considerations related to evolution and extended the disgust function into several domains: core disgust, which is disgust towards food, animals, and body products, has evolved from a system of rejection of potential toxins in food and avoidance of pathogens; interpersonal disgust, which aims at preserving the body, soul, and social order and stems from unwanted contact with strangers; animal nature disgust, which lies in neutralizing reminders of humans as animals; moral disgust, which protects social order and derives from moral violations.

Tybur and collegues [10] improved their theory by restructuring it in the form of a functional model. It provided a better interpretation of those domains (pathogen disgust) that contribute to the perception of disgust, with the exception of sex and moral offences, and infection. Pathogen disgust is the natural evolutionary outcome that leads to the avoidance of pathogens, determined by objects that have a fair chance of containing infectious agents, such as rotting food and body fluids, such as spit or vomit. Any stimulus that activates one of our five senses, if perceived as pathogenic or infected, will cause the individual to reject it and withdraw [20]. In addition to pathogenic aspects, various studies have highlighted how the refusal/avoidance of food is also linked to customs and cultural background together with individual characteristics [10,21]. Accordingly, the RHM functional model added moral disgust which assesses the consumption of certain animal-based dishes as appropriate and inappropriate. For example, many people in Western cultures would consider the idea of eating dogs and cats as disgusting despite the fact that it is a typical dish of some Eastern cultures [12]. Furthermore, a large number of people decide to avoid consuming animal-based foods or foods derived from them because of new technologies used in the food sector [22] or due to the procedures for the treatment of meat which are considered by vegetarians and vegans as disgusting, leading them to the avoidance of such foods [23]. It has been noticed that the presence of ecological pathogens is linked to groups’ morals [24,25].

Although the moral aspect conditions the consumer’s behavior, we should also consider the non-moral component that is involved in subjective decisions [16]. It has been found that food disgust is determined by the individual’s intention to pursue social norms and roles through the maintenance of identity [26]. Researchers noticed that areas where individuals perceive an increased risk of infection lead them to adapt their decision-making processes in relation to greater disgust [27]. Pellegrino, Crandall, and Seo [28] found that there were differences in hygiene and hand washing behavior between Hispanics and Caucasians depending on the type of food being handled. La Barbera and colleagues [29] conducted a study that verified the forms of disgust and neophobia towards food based on insects. It was found that the presence of implicit positive attitudes in individuals led to less disgust towards the consumption of insect-based foods. This shows how individual characteristics and culture play a key role in the decision-making process of consumption/rejection of a new or disgusting food evaluated on a moral, infectious, cultural, and behavioral basis.

## 3. Aim of Study

As it was stated that food disgust is a food rejection emotion aimed at avoiding potentially noxious substances [30], previous studies have demonstrated that some stimuli are associated with the presence of bacteria, viruses, or other microorganisms that can cause disease, while others are related to cultural aspects [12]. Therefore, considering that, as stated above, the refusal/avoidance of food is linked to customs and cultural background together with individual characteristics [10,21], we can hypothesize that individual characteristics could be related to the choice or the avoidance of the food. In detail, the present study has the aim to deepen the relationship between vulnerability to disease and food neophobia, with food disgust, hypothesizing that both could affect the rejection of some food characteristics. Therefore, we hypothesize that:

**Hypothesis** **1** **(H1).**  *Food neophobia affects food disgust directly.*

**Hypothesis** **2** **(H2).**  *Food neophobia affects food disgust by the mediation of perceived infectability.*

**Hypothesis** **3** **(H3).**  *Food neophobia affects food disgust by the mediation of germ aversion.*

**Figure 1:**
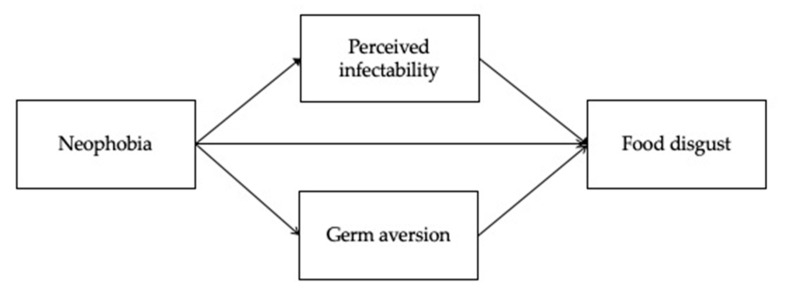
The hypothesized model.

The hypothesized model is represented in Figure 1.

## 4. Materials and Methods

### 4.1. Measures

The scales are reported in Appendix A.

The Food Neophobia Scale (FNS; Table A1) [3,31] is a psychometric scale to measure food neophobia. Respondents are requested to indicate their degree of agreement/disagreement with 10 statements about foods or eating situations using a 7-point Likert scale (from strongly agree to strongly disagree). The Cronbach’s alpha of the FNS in the sample of the study is 0.84.

Perceived Vulnerability to Disease (PVD; Table A2) [32] assesses the beliefs about personal susceptibility to the transmission of infectious diseases and emotional discomfort in the presence of potential disease transmission through a 15-item questionnaire with a 7-point Likert scale (from strongly agree to strongly disagree); it is composed of two dimensions: perceived infectability measures beliefs about immunological functioning and personal susceptibility to infectious diseases (Cronbach’s alpha = 0.77 in the sample of the study); germ aversion measures aversive affective responses to situations that connote a relatively high likelihood of pathogen transmission (Cronbach’s alpha = 0.69 in the sample of the study). The Cronbach’s alpha of the whole scale calculated for the sample of the study is 0.72.

The Food Disgust Scale (FDS; Table A3) [12] is a psychometrically validated scale composed of 32 items that measure food disgust as a measure of trait disgust (i.e., a person’s emotional predisposition to be more or less easily disgusted by certain food-related cues). The Cronbach’s alpha calculated for the sample of the study is 0.93.

### 4.2. Participants

The recruitment of the participants used convenience sampling, on a voluntary basis from the general population. The online survey was administered individually and anonymously in the period between April 2019 and December 2020; the participants gave their consent for participation before starting and could interrupt it at any moment. The participants were chosen on the basis of proximity to the researchers and their collaborators. In addition, eating disorders were used as an exclusion criterion. They received the following instructions: “We are conducting a study on attitudes towards particular, and often new types of food consumption. Below you will be given the opportunity to express your opinion on some aspects of your attitude towards such consumption. Please respond sincerely as there are no right or wrong answers, but answers more or less close to your opinion. The questionnaire is absolutely anonymous and the data collected will be used in aggregate form for statistical purposes only. Thanks for your collaboration”.

The respondents were 487 Italian adults (220 males, 45.2%; 267 females, 54.8%) aged from 18 to 84 years (M = 35.22; SD = 14.06). We contacted 550 participants but 13 of them (2.36%) met the exclusion criteria, so were excluded; then, 50 (9.31%) did not complete the survey, so were excluded from the data analyses. Almost one third of them had a university degree (150, 30.8%) and high school degree (136, 27.9%); the remaining participants had a lower level of education (201, 41.3%). The respondents’ working activities were distributed as follows: students (134, 27.5%), teachers (52, 10.7%), employees (98, 20.1%), entrepreneurs/freelancers (69, 14.2%), and others (134, 27.5%). The Ethics Commission of the university reviewed and approved the survey.

### 4.3. Data Analyses

A path analysis was carried out to test the hypothesized model using Lisrel 8.80 [33]. The goodness-of-fit indices provided by Lisrel include chi-square (χ^2^), comparative fit index (CFI), the root mean square error of approximation (RMSEA), and the standardized root mean square residual (SRMR). A significant χ^2^ value leads to the rejection of the null hypothesis that the model fits in the population. The CFI provides an evaluation of the difference between an independent model and the specified model. For the CFI, values over 0.90 suggest an acceptable fit, while values over 0.95 suggest a good fit [34]. According to Browne and Cudeck [35] (1993), an RMSEA < 0.09 is still an indicator of a reasonable error of approximation in smaller samples.

The significance of the indirect effects was calculated using the SPSS Process v.3.4 macro [36], through the bootstrapping method with 5000 repetitions and a confidence interval (CI) of 95%. Other well-known analytical tools, such as correlations, were also used, implemented using SPSS 25.0 (IBM, Armonk, New York, USA).

## 5. Results

### 5.1. Descriptive Statistics and Correlations

The mean, standard deviation, and correlations are presented in Table 1. The results demonstrate that perceived infectability is significantly correlated to food neophobia and food disgust; food disgust and food neophobia are significantly and strongly associated. Germ aversion does not have significant correlations with the other variables.

### 5.2. Path Analysis and Mediational Analysis

**Figure 2:**
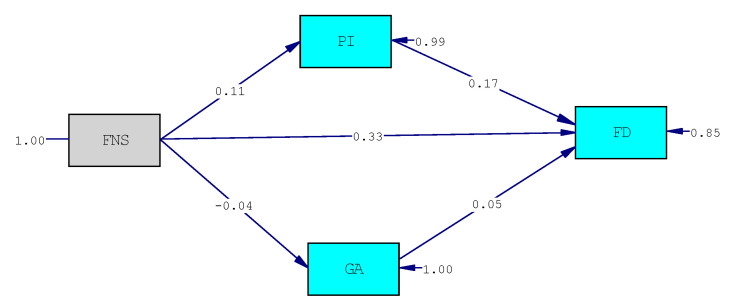
The final model. Chi-Square = 0.01; df = 1; *p*-value = 0.91495; RMSEA = 0.000. FNS = food neophobia. PI = perceived infectability. GA = germ aversion. FD = food disgust.

We tested our hypotheses using structural equation modeling analysis. The main fit indices suggest excellent fit indices (χ^2^_(1)_ = 0.011, *p* = 0.91; CFI = 1; RMSEA = 0.0; SRMR = 0.002). The final model is presented in Figure 2. The significant relationships are indicated by standardized β, that represents the intensity of the effect of the predictor on the outcome; as reported in Figure 2, both the direct relationship between food neophobia and food disgust and the indirect one, through the mediation of the perceived infectability, are significant, confirming hypotheses 1 and 2; on the contrary, there are no significant relationships between food neophobia and germ aversion, and the latter does not have a mediating effect in the relationship between food neophobia and food disgust; so hypothesis 3 cannot be confirmed.

The mediational hypothesis was tested through the verification of the significance of the indirect effects using the bootstrapping method. Table 2 reports the results of the mediations, presenting the β values, which indicate the intensity of the effect, and the 95% confidence intervals (CIs), which indicate the significance of the effect with a 5% probability of error (CIs that do not include 0 are significant). The results, presented in Table 2, show that food neophobia directly affects food disgust (β = 0.33, *p* < 0.001), confirming hypothesis 1; moreover, the path from neophobia to perceived infectability (β = 0.11, *p* < 0.001) is significant, as well as the path from perceived infectability to food disgust (β = 0.17, *p* < 0.01), showing both a direct and indirect effect of neophobia on food disgust (IE = 0.06; CI 0.009–0.12), mediated by perceived infectability (these results confirm hypothesis 2). 

On the contrary, neither the path from neophobia to germ aversion nor the path from germ aversion to food disgust is significant, so the indirect effect of neophobia on food disgust, through the mediation of germ aversion, was not found, thus rejecting hypothesis 3.

## 6. Discussion

The aim of the present study was to verify the relationship between food neophobia and food disgust, analyzing the potential mediational role of vulnerability to disease. The research hypotheses are partially confirmed; among the two dimensions of vulnerability to disease, perceived infectability was found to play a mediational role in the relationship between food neophobia and food disgust; germ aversion did not have significant relations with the two constructs. Moreover, a direct effect of food neophobia, defined as a strong bias toward novel food avoidance [31], on food disgust was found.

Even though several studies have shown that food neophobia is closely related to food disgust [2,15,16], only a few studies have measured the relationship between food neophobia and disgust and have explored the hypothesis that individual differences in disgust are associated with individual differences in food neophobia [2,37]; only recently was food disgust sensitivity found to be one influential factor in food neophobia [38]. 

Regarding the relationships between food neophobia, vulnerability to disease, and food disgust, in our literature review, we did not find any previous study deepening the role of vulnerability to disease in the relationship between the two cited constructs.

As the relationship between the perception of vulnerability to disease could lead to people’s sensitivity to all types of stimuli perceived as unpleasant [17], food disgust can be considered as an outcome of this sensitivity toward unpleasant stimuli. The few studies that have related food disgust to vulnerability to disease show similar results; Curtis, De Barra, and Aunger [39] found that the dimensions of perceived vulnerability to disease, perceived infectability, and aversion to germs correlated with two different disgust sensitivity measures, showing that the disgust system is affected by previous states of illness. Moreover, in Hartmann and Siegrist’s [12] and in Egolf et al.’s [37] studies, food disgust and germ aversion were highly correlated. Surprisingly, in our study, only perceived infectability is related to food disgust, playing a mediational role between food neophobia and food disgust; germ aversion does not have a similar role. Although the individuals involved in the study perceive themselves to be at risk of contracting possible infections, they may not pay enough attention to the hygiene practices actually applied by themselves or in the places they visit. For example, Byrd-Bredbenner and colleagues [40] conducted a study where 97% of young adults evaluated their knowledge toward food safety as fair or adequate but 60% of them did not wash their hands properly after touching raw poultry. It is possible that faulty beliefs about how to behave in order to avoid diseases, lack of attention, or disinformation about hygiene norms may lead to a lack of germ aversion when faced with unhygienic behavior. 

Therefore, as has been stated, food disgust is a characteristic describing a person’s tendency to experience disgust toward pathogen-related food risks [36], besides the characteristics of the food, and it is also determined by some individual psychological characteristics: the temporary salience of disease, that encompasses beliefs about immunological functioning and personal susceptibility to infectious diseases [32], and individual differences in chronic concerns about disease transmission [18], also have an influence in the choice of food, and, consequently, on the variety of the diet, and the openness toward nontraditional foods. Cohen and Avieli [41] highlighted that psychological traits of individuals can lead them to be curious and interested in foreign cuisines but, at the same time, they can act as a constraint that prevents them from trying foods they have never experienced before [42]. In addition, these individual characteristics significantly influence levels of acceptance and the propensity to vary one’s diet. It has been found that insect-based, including mealworm-based, foods, when presented in familiar and recognisable forms to those in the consumer’s past experience, are more likely to be accepted, otherwise they cause disgust and prevent changes in the consumer’s dietary lifestyle [43]. Torri and colleagues [44] noted that subjective sensitivity to disgust would lead Italians to consider jellyfish consumption as inappropriate and distant from their conception of traditional and familiar food, which is evaluated on the basis of its habit, natural, origin, locality, processing, elaboration, and sensory features [45].

This research has fostered a greater understanding of the role played by individual beliefs of infectious vulnerability (i.e., perceived infectability) in previously unexperienced foods that generate disgust in people. Given the lack of studies investigating its function as a mediator, new research possibilities are provided in order to investigate the existence of potential moderators that mitigate or reinforce the mediating effect of perceived infectability. As a result, these outcomes contribute to the practical implications of the study regarding the possibility of raising awareness in Italy through greater dissemination of the production processes and control conducted on these foods. Consequently, consumers will feel safer in consuming them rather than being sceptical because of their beliefs that lead them to consider such dishes as noxious, infectious, and disgusting at the mere thought of consuming them. Spreading the benefits through existing communication channels such as social media could increase consumer knowledge and curiosity to such an extent that it would also stimulate companies to invest more in these foods and deal with the socio-cultural and practical issues related to them. 

## 7. Limitations and Conclusions

The considerations set out in the discussion show us how current food consumption trends are moving towards sustainable, healthy food production that is strongly linked to local territories. These habits could soon become part of people’s daily lives, just as sustainability could also be recognized in products originating from geographically distant realities. In this last hypothesis, however, a series of ready variables can intervene that push towards rejection if not towards a real disgust towards new foods. To better understand the reasons that influence resistance to the consumption of alternative foods, it therefore becomes necessary to investigate the functions that play a role in influencing both individual and social variable choice processes. In the first case, the perception of vulnerability to disease plays a role. In the second case, some marketing strategies aimed at reinforcing hatred or love towards certain eating styles play a role [46,47].

Given this premise, the results of the study must be read in light of some limitations of the research: the study was conducted using convenience samples, which do not guarantee the representativeness of the sample; moreover, the transversality of the investigation did not allow us to investigate the causal relationships between the variables. Future research should involve a larger number of participants, selected through population stratification, using a longitudinal research design. However, despite these limitations, these results provide some important suggestions for further research on this topic. Firstly, the results presented are based on data collected before the start of the pandemic, so they are neutral with respect to the effects caused by the ongoing event; second, future studies should investigate the controversial role of germ aversion in influencing food disgust; finally, considering the role played by individual psychological characteristics in food choices, future studies could explore the role of personality dimensions in influencing food disgust.

## Figures and Tables

**Table 1:** Descriptive statistics and correlations between the studied variables.

	M	SD	1	2	3	4
1. Perceived infectability	27.39	5.92	1			
2. Germ aversion	28.41	4.05	0.001	1		
3. Food neophobia	27.94	10.28	0.11 *	−0.04	1	
4. Food disgust	122.24	29.96	0.21 ***	0.04	0.35 ***	1

Note: * *p* < 0.05; *** *p* < 0.001.

**Table 2:** Effects of neophobia on food disgust through perceived infectability and germ aversion (β).

Paths	Indirect Effect		Direct Effect		Total Effect	
	β	C.I. 95%	β	C.I. 95%	β	C.I. 95%
Food neophobia–Perceived infectability–Food disgust	0.06	0.009–0.12	0.96	0.72–1.20	1.02	0.77–1.26
Food neophobia–Germ aversion–Food disgust	−0.006	−0.03–0.09	1.02	0.78–1.27	1.02	0.77–1.26

## Data Availability

Data are available upon request to the corresponding author

## Appendix A. The Research Survey

**Table A1:** Food Neophobia Scale (FNS, Pliner and Hobden, 1992; Ritchey et al., 2003); in brackets: Italian translation.

I am constantly sampling new and different foods. (Assaggio frequentemente cibi nuovi e diversi) *	1	2	3	4	5	6	7
I do not trust new foods. (Non mi fido dei nuovi alimenti)	1	2	3	4	5	6	7
If I do not know what is in a food, I will not try it. (Se non conosco un particolare cibo, non lo provo)	1	2	3	4	5	6	7
I like foods from different countries. (Mi piacciono gli alimenti di culture diverse) *	1	2	3	4	5	6	7
Ethnic food looks too weird to eat. (Il cibo etnico mi appare troppo strano da mangiare)	1	2	3	4	5	6	7
At dinner parties, I will try a new food. (Nei party a cui sono inviato, provo spesso cibi nuovi) *	1	2	3	4	5	6	7
I am afraid to eat things I have never had before. (Ho paura di mangiare cose che non ho mai provato prima)	1	2	3	4	5	6	7
I am very particular about the foods I will eat. (Sono molto pretenzioso riguardo ai cibi che mangio)	1	2	3	4	5	6	7
I will eat almost anything. (Mangio quasi tutto) *	1	2	3	4	5	6	7
I like to try new ethnic restaurants. (Mi piace provare nuovi ristoranti etnici) *	1	2	3	4	5	6	7

* Item with reverse score.

**Table A2:** Perceived Vulnerability to Disease (PVD, Duncan et al., 2009); in brackets: Italian translation.

It really bothers me when people sneeze without covering their mouths. (Mi disturba fortemente quando le persone starnutiscono senza coprirsi la bocca)	1	2	3	4	5	6	7
2. If an illness is ‘going around’, I will get it. (Se c’è “in giro” una malattia la prenderò)	1	2	3	4	5	6	7
3. I am comfortable sharing a water bottle with a friend (Non ho alcun problema a condividere una bottiglia d’acqua con un amico) *	1	2	3	4	5	6	7
4. I do not like to write with a pencil someone else has obviously chewed on. (Non mi piace utilizzare una penna che qualcun altro ha precedentemente masticato)	1	2	3	4	5	6	7
5. My past experiences make me believe I am not likely to get sick even when my friends are sick. (In base alla mia esperienza sono portato a credere che non sono posso ammalarmi anche quando i miei amici sono malati) *	1	2	3	4	5	6	7
6. I have a history of susceptibility to infectious disease. (Ho una storia personale di particolare sensibilità alle malattie infettive)	1	2	3	4	5	6	7
7. I prefer to wash my hands pretty soon after shaking someone’s hand. (Preferisco lavarmi le mani immediatamente dopo aver stretto la mano a qualcuno)	1	2	3	4	5	6	7
8. In general, I am very susceptible to colds, flu and other infectious diseases. (In generale, sono molto sensibile a raffreddori, influenze e altre malattie infettive)	1	2	3	4	5	6	7
9. I dislike wearing used clothes because you do not know what the last person who wore it was like. (Non mi piace indossare abiti usati perché non conosco lo stato di salute di chi li ha indossati precedentemente)	1	2	3	4	5	6	7
10. I am more likely than the people around me to catch an infectious disease. (Tra le persone che frequento sono il più predisposto a prendere una malattia infettiva)	1	2	3	4	5	6	7
11. My hands do not feel dirty after touching money. (Non avverto un senso di sporcizia nelle mani dopo aver toccato i soldi) *	1	2	3	4	5	6	7
12. I am unlikely to catch a cold, flu or other illness, even if it is ‘going around’. (È improbabile che prenda il raffreddore, l’influenza o altre malattie, anche negli abituali periodi di contagio) *	1	2	3	4	5	6	7
13. It does not make me anxious to be around sick people. (Non mi trasmette ansia frequentare persone malate) *	1	2	3	4	5	6	7
14. My immune system protects me from most illnesses that other people get. (Il mio sistema immunitario mi protegge dalla maggior parte delle malattie che gli altri abitualmente prendono) *	1	2	3	4	5	6	7
15. I avoid using public telephones because of the risk that I may catch something from the previous user. (Evito di utilizzare i telefoni pubblici per evitare il rischio di contagio da chi li ha utilizzati precedentemente)	1	2	3	4	5	6	7

* Item with reverse score.

**Table A3:** Food Disgust Scale (FDS, Hartmann & Siegrist, 2018); in brackets: Italian translation.

To put animal cartilage into my mouth (Sentire in bocca del grasso animale)	1	2	3	4	5	6
2. To see raw meat (Vedere sul bancone carne cruda)	1	2	3	4	5	6
3. To eat a steak that is still bloody inside (Mangiare una bistecca al sangue)	1	2	3	4	5	6
4. To see a whole pig en brochette (Vedere un maialino intero allo spiedo)	1	2	3	4	5	6
5. To eat with dirty silverware in a restaurant (Pensare di mangiare in un ristorante con posate sporche)	1	2	3	4	5	6
6. A meal prepared by a cook who has greasy hair and dirty fingernails (Immaginare che il cibo sia preparato da un cuoco con i capelli unti e le unghie sporche)	1	2	3	4	5	6
7. If the cook in a restaurant has an open cut (Vedere lo chef con un taglio al dito non fasciato e leggermente insanguinato)	1	2	3	4	5	6
8. If people blow their nose before they serve my meal (Vedere chi mi serve il cibo soffiarsi poco prima il naso)	1	2	3	4	5	6
9. Another person’s hair in my soup (Vedere un capello nella zuppa)	1	2	3	4	5	6
10. Food donated from a neighbor whom I barely know (Non conoscere bene il cibo che mi ha offerto un vicino)	1	2	3	4	5	6
11. If a friend bites into my bread (Vedere un mio commensale mordere il mio pane)	1	2	3	4	5	6
12. To drink from the same drinking glass a friend has already drunk from (Bere dallo stesso bicchiere in cui ha bevuto un mio commensale)	1	2	3	4	5	6
13. If friends or acquaintance have touched my food (Immaginare che i miei commensali che toccano il mio cibo)	1	2	3	4	5	6
14. To eat the mold-free part of a moldy tomato (Mangiare la parte senza muffa di un pomodoro andato a male)	1	2	3	4	5	6
15. To eat bread from which mold was cut away (Mangiare del pane da cui è stata tagliata la parte ammuffita)	1	2	3	4	5	6
16. To eat hard cheese from which mold was cut off (Mangiare del formaggio dal quale è stata precedentemente tolta la muffa)	1	2	3	4	5	6
17. To eat marmalade from which mold was removed from the surface (Mangiare della marmellata che aveva della muffa sulla superficie)	1	2	3	4	5	6
18. To eat overripe fruits (Mangiare frutta troppo matura)	1	2	3	4	5	6
19. To eat a banana that has black spots (Mangiare una banana con troppe macchie nere)	1	2	3	4	5	6
20. To eat fruits (e.g., apple and peach) with pressure marks (Mangiare della frutta (ad es.: mela, pesca) con ammaccature)	1	2	3	4	5	6
21. To eat apple slices that turned brown when exposed to air (Mangiare pezzi di mela eccessivamente coloriti perché esposti all’aria)	1	2	3	4	5	6
22. To have a whole fish with its head on the plate (Avere servito sul piatto un pesce intero compresa la testa)	1	2	3	4	5	6
23. To eat raw fish like sushi (L’idea di mangiare pesce crudo (ad es.: sushi o marinato)	1	2	3	4	5	6
24. The smell in a fish shop or in fish sections with fresh fish (L’odore in una pescheria o nel reparto di pesce fresco in un supermercato)	1	2	3	4	5	6
25. The texture of some kinds of fish in the mouth (La particolare consistenza in bocca di alcune specie di pesci e/o frutti di mare)	1	2	3	4	5	6
26. To eat brown-colored avocado pulp (Mangiare da un avocado la polpa eccessivamente colorita)	1	2	3	4	5	6
27. To eat an overripe cucumber that can already be bent (Mangiare un cetriolo eccessivamente molle)	1	2	3	4	5	6
28. To eat shrunken radishes (Mangiare dei ravanelli troppo friabili)	1	2	3	4	5	6
29. To eat salad that is not crispy anymore (Mangiare dell’insalata non più croccante)	1	2	3	4	5	6
30. There is a maggot in the cherry that I wanted to eat (Vedere una larva in una ciliegia che sto per mangiare)	1	2	3	4	5	6
31. There is a little snail in the salad that I wanted to eat (Vedere una piccola lumaca nella mia insalata)	1	2	3	4	5	6
32. There is a worm in my apple (Vedere un verme in una mela)	1	2	3	4	5	6
